# The effect of a single visit to a health coach on perceived health in 50-year old residents in a high-income country – a randomised controlled trial

**DOI:** 10.1080/02813432.2022.2057035

**Published:** 2022-04-01

**Authors:** Sofia Ryd, Gerth Persson, Ronny Kent Gunnarsson

**Affiliations:** a General Practice/Family Medicine, School of Public Health and Community Medicine, Institute of Medicine, the Sahlgrenska Academy, University of Gothenburg, Gothenburg, Sweden; b Gert Persson Läkarkonsult, Västra Götalands län, Sweden; c Research, Development, Education and Innovation, Primary Health Care, Region Västra Götaland, Sweden

**Keywords:** Health coaching, health promotion, health behaviour, quality of life, primary prevention

## Abstract

**Objective:**

To evaluate the one-year-effect of a single visit to a health coach on perceived health and exercise level in 50-year-old citizens.

**Design:**

One factor design randomised controlled trial.

**Setting:**

Participants were randomly selected from the Swedish Population Register.

**Subjects:**

50-year-old residents of the town of Alingsås, Sweden (*n* = 105).

**Intervention:**

The intervention group (*n* = 52) received a single one-hour visit to a health coach. The control group (*n* = 53) received no intervention.

**Main outcome measures:**

Change over 12 months in the SF-36 dimensions physical functioning, role-physical, bodily pain, general health, vitality, social functioning, role-emotional, mental health, physical component summary and mental component summary. Reported health transition at follow-up. Change in exercise level.

**Results:**

The control group changed their perceived health more favourable than the intervention group in the following dimensions of the SF-36; general health (*p* = 0.0055–0.025), role-emotional (*p* = 0.034–0.040) and mental component summary (*p* = 0.033–0.073).

**Conclusion:**

A single visit to a health coach does not improve perceived health or exercise-level in 50-year-old citizens. On the contrary it may make perceived health worse.

**Key points**
- Research on health coaching has emerged in the last 20 years, but is diverse and the characteristics of a successful health coaching intervention are still unknown.
- There is a lack of randomised controlled trials evaluating long-term effectiveness of health coaching.
- This randomised controlled trial concludes that a single visit to a health coach does not improve, but rather impairs, perceived health in 50-year olds.

## Introduction

Although a large proportion of Europeans perceive their health as good, the prevalence of chronic conditions and lifestyle-related conditions is increasing, and the burden of poor mental health is high. Therefore, more primary prevention and better access to health care have been requested [1]. Primary health care (PHC) is often the first access point to health care and an important provider of primary prevention. However, several countries have lacking resources for primary prevention within PHC and there is a need to investigate alternative ways of delivering primary prevention in order to improve people’s health [2,3]. Health coaching is an emerging field that has successfully been used in primary prevention and promoting self-management of several chronic health conditions [4]. However, most studies on health coaching have been made on extensive health coaching interventions with multiple sessions [5]. Since there is a need for cost-effectiveness in PHC, research is needed to investigate if a single visit to a health coach could be beneficial.

### Perceived health

Low perceived health-status has been shown as a strong predictor of future mortality [6]. This statistical association persists when health-related factors such as chronic disease are controlled for [7]. This may be explained by a correlation between physical health and mortality. Another possibility is that this association is due to factors that are not related to physical health but rather to health behaviour, individual resources and personality traits. A third possibility is that it is a statistical artefact due to limitations in the number of indicators used in population studies. Regardless of the cause of this association, perceived health is widely considered as a useful predictor of mortality and often used as a measurement in population studies [6].

It has been shown that perceived health is negatively associated with health factors such as smoking, high BMI and low exercise level [8,9]. These factors can be altered through lifestyle changes, which suggests that perceived health can be improved through lifestyle interventions that target these factors.

### Primary health care

As the population grows older, the total burden of illness increases and more resources for health care are required. Consequently, there is a growing need for PHC and GPs in many high-income countries. However, several countries including Sweden already have a shortage of GPs [10–12].

The Swedish Agency for Health and Care Services Analysis recently published a report based on results from an international survey of primary care doctors in 11 countries [13]. The report concludes that 60% of GPs in Sweden report their work as very or tremendously stressful. High workload and insufficient time allocated for each patient are mentioned as the main causes of stress. Therefore, the Swedish Agency for Health and Care Services Analysis recommends that the possibility of relocating some tasks from GPs to other occupational categories should be further investigated. Hence, a complementary solution is to try and reduce the need for health care by increasing efforts to deliver primary prevention.

### Health coaching

There is no clear definition of the term ‘health coach’, but it is referred to in the literature as a person who is trained and certified in safely guiding patients or clients in health behaviour change [14]. Health coaches are used in various settings for widely different purposes ranging from workplace health promotion to diabetes self-care and cancer pain management [15–17]. Although there are multiple health coaching models and theories (e.g. transtheoretical model of change, social cognitive theory, motivational interviewing), all have the common goal of inducing behavioural change [18]. Studies have shown that health coaches have been successfully used in preventive care and in managing several chronic health conditions including weight loss, physical activity and diabetes management [4].

The education of health coaches is also diverse and there is no comprehensive definition or license required in order to be described as a health coach [14]. Within the health care system health coaches are often health care professionals such as nurses, dieticians, psychologists or medical assistants [18]. However, some health coaches have limited or no medical education [14].This category includes health educators, professional coaches and sometimes patients or peers [19]. Existing literature shows that health coaches working within the established health care system (nurses, etc.) are often, but not always, used in interventions targeted at chronic diseases such as diabetes and heart failure, whereas health coaches with no formal medical education are more often used within workplace health promotion and general health promotion [19–21]. Health coaches with a medical profession, usually nurses, also seem more common in the US whereas health coaches with other professions, or with no formal medical education, are more common in other countries [22].

In the context of stressed GPs and a strained PHC system, health coaches can potentially be used to meet some of patients’ needs and to improve preventive care [2]. Health coaching can improve the job satisfaction of GPs as well as decrease use of the health care system [4]. Most research has been done on health coaching in the context of chronic disease. Thus, the possible benefits of health coaching in the context of primary prevention are relatively unexplored. Furthermore, the research on health coaching in a preventive setting has mainly been conducted in a workplace setting.

Most research on successful health coaching is based on longer interventions with multiple sessions where the coach creates a relationship with their client or patient [5]. These long interventions include frequent follow-ups with the health coach, which means monetary as well as timely costs. More research is needed on the possible effect of briefer interventions that might be even more cost-effective. Furthermore, a lack of randomised controlled trials has also been identified [23].

The primary aim of this randomised controlled trial is to evaluate the one-year-effect of a single visit to a health coach on perceived health among 50-year-old citizens. The secondary aim is to evaluate the 1-year-effect of a single visit to a health coach on exercise level in 50-year-old citizens.

## Material and methods

The study was a one factor design randomised controlled trial. Randomisation, intervention and one-year follow-up took place between the years 2000 and 2002.

### Study population and sample size calculations

Half of the 50-year old residents in Alingsås, a town in the south-west part of Sweden were randomly selected from the Swedish Population Register and invited to participate (*n* = 135). The population size was based on statistical power calculations from the SF-36 manual, where a sample size between 34 and 118 per group is needed in order to detect a 10-point difference between groups in SF-36 dimensions in a two-tailed test (statistical power of 80%, and level of significance set to 0.05) [24]. For practical reasons the compromise of 67 participants in each group was chosen as this would be sufficient to find a 10-point difference in six of the eight SF36 dimensions.

### Data collection

A two-piece survey was sent out to all study participants (Appendices 1 and 2). The first part of the survey consisted of the validated SF-36 health survey questionnaire version 1, which is a validated questionnaire that has been widely used in research. SF-36 measures perceived health on eight specific multi-item dimensions and two main dimensions. The specific dimensions are *physical functioning, role-physical, bodily pain, general health, vitality, social functioning, role-emotional* and *mental health*. These eight dimensions can be compiled into two main dimensions; *mental component summary* and *physical component summary*. The SF-36 questionnaire also contains an independent item that measures reported health transition in the last year [25].

The second part of the survey was a non-validated questionnaire containing questions about gender, marital status, country of birth, country of birth of parents, current and former smoking habits, number of visits to health care in the preceding 12 months, reasons for health care visits, which categories of health care professionals were visited, any sick leave during the preceding 12 months, main occupation, how often they engaged with relatives/friends/work mates in leisure-time, any engagement in a non-profit association and current exercise level.

The survey was distributed at baseline together with an invitation letter explaining the study, and at follow-up after approximately one year. A maximum of two additional invitations including the survey were sent in order to reach a higher response rate. Single missing answers in the SF-36 form were imputated according to the SF-36 scoring manual [25].

### Randomisation

The participants who replied to the baseline survey were block randomised into one intervention group (IG) and one control group (CG). The block size was two.

### Intervention

The intervention received by the IG consisted of a one-hour session with a health coach who conducted a Health Profile Assessment (Hälsoprofilbedömning, HPB) including a motivational conversation on health behaviour change. One single health coach was used throughout the study, who had a degree in health education as well as training in the use of HPB but otherwise no formal medical training.

HPB is a well-tried Swedish health coaching model based on a questionnaire, body measurements, blood pressure measurements, a sub maximal bicycle exercise stress test, and a discussion between the health coach and the client/patient. The assessment was made according to a standardised procedure performed by a health coach certified in HPB [26,27].

The HPB questionnaire contains lifestyle questions such as exercise-, dietary-, tobacco- and alcohol habits and health questions on perceived symptoms, current medication and perceived health. Body measurements and a sub maximal work test are used to calculate maximal aerobic capacity (l/min and ml/kg/min).

In the HPB method, the individual’s questionnaire response, measurements and exercise stress test results are used as the foundation for a conversation. This conversation is focused on building motivation for individually adapted behavioural changes with the purpose of creating a healthier lifestyle. HPB is well-researched as a screening method for individuals at risk for future illness and as a tool for creating targeted health promotion efforts [15,27,28]. The CG received no intervention.

### Statistical methods

SPSS version 25 for Windows (SPSS Inc., Chicago, IL) was used for statistical analysis. Analysis was based on survey results. Survey results from both groups at baseline and one year later was used to calculate changes in perceived health and exercise level.

Change in perceived health was calculated as difference between follow-up and baseline score in the eight specific SF-36 dimensions as well as the two main dimensions. Raw and transformed changes were used in the analysis, the latter coded as impaired (−1), unchanged (0) and improved (+1). Change in exercise level over time was calculated from a question where the participant graded their level of exercise on four levels from no exercise to hard exercise. Raw and transformed changes of exercise were used in the analysis, the latter also coded as impaired (−1), unchanged (0) and improved (+1).

Difference between groups in raw changes were analysed using independent samples t-test and Mann–Whitney *U* test as per protocol (PP), complete case (CC) and intention to treat. (ITT). Transformed changes were compared between groups using Mann–Whitney *U*-test.

The PP analysis included the participants who presented a complete set of data (baseline and follow-up survey) and only included the individuals in the IG who completed the allocated intervention. The CC analyses included all participants who presented a complete set of data including those in the IG who did not attend the intervention. The ITT analysis included all participants who were included in the randomisation process.

Missing data at baseline were replaced with study population mean in the ITT analyses. Last Observation Carried Forward (LOCF) was used to manage missing data in the follow-up surveys in the ITT analysis.

### Ethics

This study was approved by the Ethics Committee, Göteborg University (registration number 548-00). The declaration of Helsinki was adhered to. Participants were informed that participation was voluntary and that they had the right to refuse attendance without reporting any reason. Data were stored as re-identifiable with personal identity replaced by a code.

## Results

**Figure 1.**
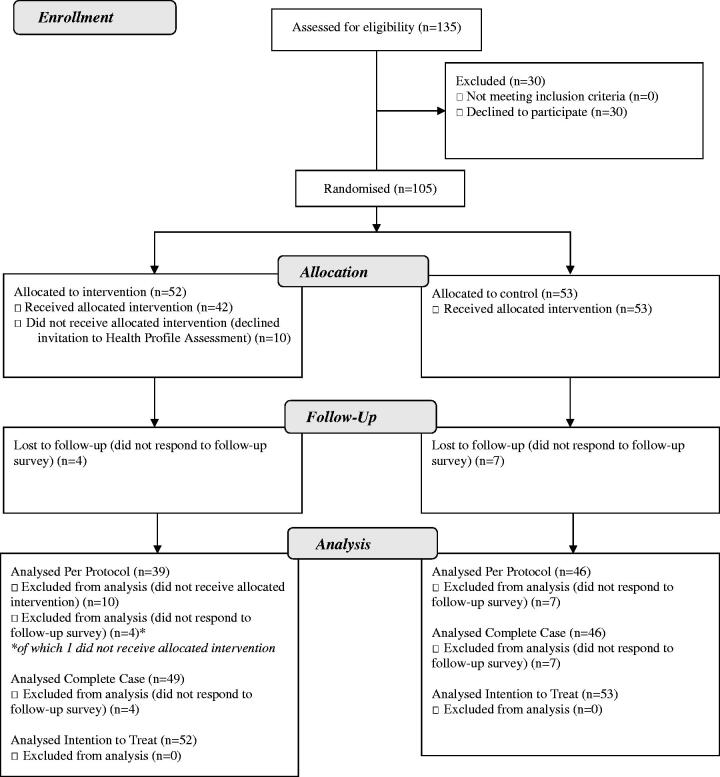
Participant flow diagram.

Of the 135 persons who were invited to participate, 105 persons responded to the baseline survey and were randomised into one IG (*n* = 52) and one CG (*n* = 53) (Figure 1). Of the 52 persons allocated to the IG, 10 declined to participate in HPB. Consequently, 42 participants conducted HPB. Four participants in the IG and seven participants in the CG did not respond to the follow-up survey.

There were no significant differences in gender, civil status, country of birth, main occupation, sick leave, smoking status, socialising habits, exercise level or BMI between the groups (Table 1). However, there was a significant difference in the degree of activity at work (*p* = 0.0041), where sedentary work was more common in the IG group (44%) than in the CG (22%) and active and physically heavy work was more common in the CG (35%) than in the IG (12%). Perceived health at baseline did not differ between the groups (Table 2) and was coherent with norm data from the Swedish population [29].

**Table 1.** Demographic characteristics, activity and smoking status of participants at baseline.

	Intervention group (*n* = 52)	Control group (*n* = 53)
Female gender % (*n*/*N*^a^)	61 (31/51)	48 (25/52)
Married or co-habitant % (*n*/*N*^a^)	78 (40/51)	87 (45/52)
Born in Sweden % (*n*/*N*^a^)	86 (44/51)	92 (48/52)
Main occupation^b^ (*n*)		
Paid work	45	45
Homemaker	0	1
Student	2	1
Unempolyed <6 months	0	1
Unemployed >6 months	1	0
Labour market measures	1	0
Early retirement/disability pension	2	4
Sick leave >6 months	2	1
Other	1	1
Degree of activity at work % (*n*/*N*^a^)		
Sedentary	44 (21/48)	22 (11/51)
Mostly sedentary	4.2 (2/48)	2.0 (1/51)
Active but not physically heavy	38 (18/48)	41 (21/51)
Active and physically heavy	15 (7/48)	35 (18/51)
Sick leave in preceding 12 months % (*n*/*N*^a^)		
0	61 (31/51)	68 (34/50)
< 1 week	20 (10/51)	22 (11/50)
1–4 weeks	8 (4/51)	6 (3/50)
> 4 weeks	12 (6/51)	4 (2/50)
Smoking status % (*n*/*N*^a^)		
Never smoked	41 (21/51)	31 (16/51)
Ex-smoker	25 (13/51)	37 (19/51)
Occasional smoker	7.8 (4/51)	12 (6/51)
Daily smoker	25 (13/51)	20 (10/51)
Socialising habits in leisure time^c^ % (*n*/*N*^a^)		
Several times a week	39 (20/51)	52 (27/52)
Several times a month	39 (20/51)	33 (17/52)
More rarely	18 (9/51)	15 (8/52)
No	4 (2/51)	0 (0/52)
Exercise level in leisure time % (*n*/*N*^a^)		
Sedentary	14 (7/51)	9.6 (5/52)
Light exercise	73 (37/51)	62 (32/52)
Regular exercise	12 (6/51)	27 (14/52)
Tough exercise or competitive sports	2.0 (1/51)	1.9 (1/52)
BMI (kg/m^2^)^d^	25 (4.0), 25 (23–27)^c^	25 (3.1), 25 (23–27)^c^
Diabetes^e^ % (*n*/*N*^a^)	2.4 (1/41)	–
Asthma^e^ % (*n*/*N*^a^)	4.9 (2/41)	–

^a^n = number of participants with characteristics. N = number of participants in the group where we have information available.

^b^More than one alternative is allowed.

^c^Do you see relatives, friends, colleagues or neighbours in your leisure time?.

^d^Mean (standard deviation), median (interquartile range).

^e^Data available for intervention group only.

**Table 2.** Perceived health of participants at baseline.

	Intervention group^a^	Control group^a^
Physical functioning^b^	90 (16), 95 (85–100)	88 (20), 95 (85–100)
Role-physical^b^	89 (24), 100 (100–100)	80 (34), 100 (75–100)
Bodily pain^b^	77 (26), 84 (64–100)	72 (27), 72 (51–100)
General health^b^	78 (23), 86 (71–97)	71 (23), 71 (57–92)
Vitality^b^	67 (26), 75 (49–85)	62 (26), 68 (36–80)
Social functioning^b^	90 (18), 100 (88–100)	85 (24), 100 (75–100)
Role-emotional^b^	87 (29), 100 (100–100)	76 (35), 100 (33–100)
Mental health^b^	80 (21), 88 (67–96)	77 (22), 84 (61–92)
Physical component summary^c^	51 (8.8), 54 (48–56)	49 (10), (44–56)
Mental component summary^d^	49 (11), 54 (43–57)	46 (12), 50 (37–55)
Reported health transition^e^	3.0 (0.34), 3.0 (3.0–3.0)	3.1 (0.65), 3.0 (3.0–3.0)

^a^Mean (standard deviation), median (interqartile range).

^b^Sub-scale/dimension of SF-36 Health Survey. Scale range: 0–100.

^c^General dimension based on sum scores of Physical Functioning, Role-Physical, Bodily Pain and General Health. Scale range: 0–100.

^d^General dimension based on sum scores of Vitality, Social Functioning, Role-Emotional and Mental Health. Scale range: 0–100.

^e^Independent question in the SF-36 Health Survey; compared to one year ago, how would you rate your health in general now? (5 = much better now than one year ago, 4 = somewhat better now than one year ago, 3 = about the same, 2 = somewhat worse now than one year ago, 1 = much worse now than one year ago).

Perceived health in the IG tended to decrease or remain unchanged in a majority of SF-36 dimensions, whereas the CG tended to improve or remain unchanged (Tables 3 and 4). Statistically significant differences (*p* < 0.05) between the groups, favouring the CG, were reached in all analyses (PP, CC and ITT) for general Health, role-emotional and mental component summary (Table 4). Change in reported health transition was significant, favouring the CG, when analysed as CC and ITT (Table 4). There was no statistically significant difference in exercise level or socialising habits between the groups (Table 4).

**Table 3.** Transformation of changes in SF-36 dimensions, exercise level and socialising habits for all participants with complete records.

	Intervention group^a^			Control group^a^		
	Decrease	No change	Increase	Decrease	No change	Increase
Physical functioning^b^	18	20	10	12	20	13
Role-physical^b^	12	31	5	7	29	10
Bodily pain^b^	12	24	12	14	17	15
General health^b^	25	11	12	15	9	21
Vitality^b^	21	4	23	18	3	25
Social functioning^b^	13	29	6	9	27	10
Role-emotional^b^	6	35	7	2	30	14
Mental health^b^	20	14	14	17	8	21
PCS^c^	29	0	19	21	0	23
MCS^d^	27	0	21	15	0	29
Exercise level	6	29	13	4	37	5
Socialising habits	7	30	11	7	32	7
RHT at follow-up^e^	13	29	6	2	37	7

^a^*n*.

^b^Sub-scale/dimension of SF-36 Health Survey.

^c^Physical Component Summary. General dimension based on sum scores of Physical Functioning, Role-Physical, Bodily Pain and General Health.

^d^Mental Component Summary. General dimension based on sum scores of Vitality, Social Functioning, Role-Emotional and Mental Health.

^e^Reported health transition. This is not a calculated change. Independent question in the SF-36 Health survey; compared to one year ago, how would you rate your health in general now? (5 = much better now than one year ago, 4 = somewhat better now than one year ago, 3 = about the same, 2 = somewhat worse now than one year ago, 1 = much worse now than one year ago).

**Table 4.** Comparison of changes in perceived health, exercise level and socialising habits between groups^a,b^.

	Per protocol analysis					Complete case analysis					Intention to treat analysis				
	IG^c^	CG^d^	T^e^	M&W^f^	MWT^g^	IG^c^	CG^d^	T^e^	M&W^f^	MWT^g^	IG^c^	CG^d^	T^e^	M&W^f^	MWT^g^
Physical functioning	−1.7	+1.5	0.25	0.33	0.45	−3.8	+1.5	0.074	0.11	0.23	−3.5	+1.5	0.056	0.084	0.17
Role-physical	−3.2	+3.8	0.29	0.40	0.46	−**11**	**+3.8**	**0.040**	0.070	0.091	−**10**	**+3.3**	**0.036**	0.068	0.086
Bodily pain	+2.6	+1.8	0.87	0.60	0.45	−2.8	+1.85	0.32	0.58	0.88	−2.6	+1.6	0.318	0.63	0.89
**General health**	−**3.8**	**+3.2**	**0.04**	**0.034**	0.083	−**6.3**	**+3.2**	**0.0063**	**0.0050**	**0.029**	−**5.8**	**+2.7**	**0.0055**	**0.0058**	**0.025**
Vitality	0.00	+5.2	0.25	0.25	0.70	−0.5	+5.2	0.20	0.18	0.57	−0.48	+4.5	0.20	0.23	0.61
Social functioning	0.00	+3.0	0.48	0.50	0.47	−1.8	+3.0	0.24	0.22	0.21	−1.7	+2.6	0.24	0.21	0.19
**Role-emotional**	**+2.6**	**+15**	**0.047**	**0.038**	**0.038**	**+0.69**	**+15**	**0.028**	**0.027**	**0.032**	**+0.64**	**+13**	**0.034**	**0.035**	**0.040**
Mental health	−0.82	+4.0	0.14	0.17	0.38	−1.8	+4.0	0.089	0.074	0.25	−1.6	+3.5	0.089	0.086	0.24
PCS^h^	−0.93	−0.28	0.69	0.77	0.43	−3.0	−0.28	0.13	0.25	0.23	−2.8	−0.085	0.094	0.17	0.17
**MCS** ^i^	**+0.44**	**+3.75**	0.078	0.090	**0.042**	**+0.39**	**+3.8**	0.072	0.088	**0.034**	**+0.36**	**+3.3**	0.073	0.064	**0.033**
Exercise level	+0.21	0.00	0.10	0.093	0.097	+0.13	0.00	0.31	0.23	0.24	+0.12	0.00	0.30	0.22	0.22
Socialising habits	+0.10	0.00	0.50	0.52	0.54	+0.17	0.00	0.28	0.43	0.48	+0.039	+0.11	0.59	0.33	0.31
RHT^j^	+3.0	+3.1	0.19	0.23	0.24	**+2.8**	**+3.1**	**0.011**	**0.020**	**0.023**	**+2.8**	**+3.1**	**0.030**	0.050	0.056

^a^Statistically significant *p*-values are bolded.

^b^Outcome variables being statically significant in all analyses (PP, CC and ITT) are bolded.

^c^Mean change in the intervention group.

^d^Mean change in the control group.

^e^Independent samples *t*-test.

^f^Mann–Whitney *U* test.

^g^Mann–Whitney *U* test using transformed data.

^h^Physical component summary.

^i^Mental component summary.

^j^Reported Health Transition, independent question from the SF-36 Health Survey Questionnaire distributed at follow-up.

## Discussion

This study could not show that a single consultation with a health coach improves perceived health or exercise level among 50-year-old citizens. On the contrary, the intervention group seemed to report worse perceived health at follow-up than the control group which indicates a potentially negative effect of a single consultation with a health coach. A possible explanation for this might be that a single consultation with a health coach informs the participant about their health and lifestyle shortcomings, but additional sessions are required to improve their situation.

### Strengths and limitations

One strength of this study is that the participants were randomly selected from the population register, eliminating the selection bias which occurs in many studies where participants are included consecutively. Another strength is that the proportion of dropouts was relatively low (19% in PP analysis and 9.5% in CC analysis). Another important strength is the randomised design with a control group.

Using the same health coach for all patients in the intervention group can be seen as a strengths when it comes to investigating a proof of concept but it may also be seen as a limitation when evaluating how this intervention would work in real life.

A limitation is that we did not collect information on patients’ expectations prior to the intervention. Hence, it is difficult to sort out if the negative effect seen in the intervention group is merely a nocebo effect or if there are true negative health effects of the intervention.

One limitation is that the sample size was slightly smaller than suggested by the sample size estimation, which hindered subgroup analysis. A larger sample would have enabled a better comparison of gender and socioeconomic differences in the effects of health coaching, which might have provided useful insights on the characteristics of those who benefit from health coaching.

The unexpected finding that a single visit to a health coach might be detrimental made us do a post hoc exploratory analysis (see supplemental material). The main findings in this post-hoc analysis firstly suggests that confounders are unlikely to explain the surprising outcome of this study. This is an expected finding in a randomised controlled trial. Second, since the *p*-values for the effect of the intervention was similar in the group comparisons and the post hoc multivariable prediction models, it implies that the unexpected negative effect of the intervention is not bound to individuals of a specific gender, smoking habit, socialising habit, exercise level or high/low BMI.

The author doing the initial planning and data collection went on to a new job position not allowing time to work on this project. Data have since then been stored in a locked cabinet until another person was found that could take on the remaining work. Hence, another limitation is that the 50-year-olds of 20 years ago might not be representative of the 50-year-olds of today. Health attitudes and knowledge changes over time and it is possible that the general population today would react differently to messages provided by the health coach.

### Findings in relation to other research on health coaching

The health coaching intervention used in this randomised controlled trial, HPB, was designed and has been studied as a motivator for positive health behavioural change and as a screening method for future illness [27,30]. HPB is currently used within occupational health care as a screening instrument for targeted workplace health promotion [31]. There are established associations between an ‘unhealthy’ HPB profile and cardiovascular morbidity, overall mortality and high sick leave [28]. However, there are no studies on the efficacy of HPB alone in changing participants’ health behaviour, exercise level or perceived health. The developers of the method have discussed that follow-up and other efforts are needed in addition to HPB to induce health behaviour change [30]. This study appears to be the first randomised controlled study evaluating the effects of HPB.

The research field of health coaching in general has grown tremendously in the last decades [19]. However, it is still difficult to generalise the characteristics of successful health coaching interventions since research methods on the topic have been diverse regarding coaching methods as well as outcome measures [5]. Most studies are targeted at chronic conditions and/or were not conducted within a PHC setting [20,23]. Some brief interventions report positive outcomes [17,32–34], yet the components leading to success are hard to establish since the studies are difficult to compare. The positive effects of brief health coaching interventions are often small and, in some cases not significantly different from simply giving patients written educational material [35,36]. Studies measuring the effects of a single visit to a health coach does not seem to have been done before.

Some studies have shown that health coaching is more effective on individuals with low perceived health at baseline [37,38]. In addition, research that report positive outcomes often only include participants with known chronic conditions, high BMI or cardiovascular risk factors [16,17,33]. Since the participants of our study were a random sample of 50-year-olds who generally perceived their health as good at baseline (Table 2) and only a small proportion of the intervention group had known chronic conditions such as asthma or diabetes (Table 1), they might not be the optimal candidates for health coaching.

Our research is in line with Leveille et al. [39] who found no significant effects on general health or quality of life in a study on internet based coaching in a PHC setting. The study utilised a nurse health coach who briefly communicated with patients through an internet platform to promote communication between the participant and their physician. Glasgow et al. [40] also failed to find positive effects on quality of life in a randomised controlled trial with two sessions of health coaching. The study population consisted of patients with type 2 diabetes in a PHC setting. A possible explanation might be that these brief interventions, similarly to the intervention of our study, are enough to inform the patient about their health shortcomings, but too short to induce behavioural change or improve perceived health.

Even more in line with the findings of our study, Shah et al. [22] found a small negative effect on psychological wellbeing when evaluating a PHC health coaching programme. In this study, PHC patients with chronic disease were directed to a non-medical health coach instead of their GP. This suggests that re-directing PHC patients from professionals with medical training to those who are less qualified is not an effective way of improving health outcomes.

### Conclusions and implications

The primary aim of this study was to evaluate the 1-year effect on perceived health of a single consultation with a health coach among 50-year-old citizens. The main conclusion is that a single consultation with a health coach is not sufficient to improve perceived health or exercise level in healthy middle-aged adults. It might even worsen the perceived health. However, it is difficult to conclude the reason why this intervention failed to improve perceived health or exercise level since existing research on the topic is inconsistent and interventions are difficult to compare. Future preventive efforts within PHC should focus on more extensive health coaching interventions with multiple sessions over time.

## Supplementary Material

Supplemental Material
