# Supplementary material for: The effect of a single visit to a health coach on perceived health in 50-year old residents in a high-income country – a randomised controlled trial
Source: Scand J Prim Health Care. 2022 Apr 1;40(1):129–38. doi: 10.1080/02813432.2022.2057035 (PMC9090358; doi:10.1080/02813432.2022.2057035)
Supplement: Supplemental Material [file IPRI_A_2057035_SM3069.docx]

**Supplemental material – Post hoc exploratory analysis**

The finding that a health coach impairs perceived health was very surprising. Hence, an additional post hoc statistical analysis was made to explore patient factors at baseline associated with change in perceived health-related quality of life.

**Methods**

Twelve multivariable binary logistic regression models were created with change in each dimension of SF-36, exercise level and socialising habits in leisure time as dependent outcome variables. The dependent variable was coded as follows: worsening or no change coded as 0 while improvement was coded as 1. Gender, smoking status at baseline, socialising habits at baseline, exercise level at baseline, BMI at baseline and group allocation were used as independent variables.

Exercise level at baseline was coded as follows: “sedentary” = 1, “light exercise” = 2, “regular exercise” = 3 and “tough exercise or competitive sports” =4. Smoking status at baseline was coded as follows: “never smoked” =1, “ex-smoker” = 2, “occasional smoker” = 3 and “daily smoker” =4. Hence, the outcome is odds ratio for an increase in smoking status of one step. Socialising habits in leisure time at baseline was coded as follows: “”no” = 1, “more rarely” = 2, “several times a month” = 3, “several times a week” = 4. Similarly, the outcome is odds ratios for an increase in socialising habits in leisure time of one step.

Since information on diabetes, asthma, systolic and diastolic blood pressure and oxygen uptake (ml/kg/min) at baseline was only known for the intervention group, these independent variables were not included as independent variables. ROC curve analysis was used to internally validate the prediction models and only models statistically different from random variation are presented.

**Results**

The models for change in role-physical, general health, social functioning, role-emotional, mental health, physical component summary, mental component summary and exercise level had areas under curve significantly higher than 0.5 in the ROC analysis (p<0.05) (Supplemental table 1). Hence, eight of the twelve models could predict the outcome better than pure chance (Supplemental table 2).

Female gender was significantly associated with an increase in exercise level (p=0.031, aOR 7.2, 2.2-43 95% CI) (Supplemental table 3). Frequent smoking was independently significantly associated with improvement in role-emotional (p=0.035. aOR 1.7, 1.0-2.7 95 % CI) (Supplemental table 3). An active social life was independently significantly associated with less probability of improving in mental component summary (p=0.049, aOR 0.5, 0.25-1.0 95 % CI) (Supplemental table 3). A high exercise level at baseline was independently significantly associated with no increase in exercise level (p=0.0024, aOR 0.10, 0.021-0.43 95 % CI) (Supplemental table 3).

In supplemental table 2 we see that for role emotional (RE) and mental component summary (MCS) the group allocation is statistically significant (or for RE almost significant) at the same time as another variable is statistically significant. Further exploring interactions between these shows that there is no significant interaction between increasing smoking and group allocation for RE as the outcome variable (p=0.42). Neither is there any interaction between socialising habits at baseline and group allocation for MCS as the outcome variable (p=0.96)

**Interpretation**

The finding that a high baseline exercise level is associated with no increased exercise is expected since a high exercise level leaves little room for further improvement. Similarly, an active social life at baseline is associated with less chance for improvement in the mental component summary because they are already good at baseline leaving little room for further improvement.

The finding that female gender independently was significantly associated with an increase in exercise level and that frequent smoking was significantly associated with improvement in role-emotional are new. However, it was outside the scope of this study to further speculate on possible reasons for this.

The main finding that seeing a health coach impairs perceived health related quality of life remains after this post-hoc analysis (table 4 and supplemental table 2 and 3). The findings in this post-hoc analysis firstly suggests that confounders are unlikely to explain the surprising outcome of this study. This an expected finding in a randomised controlled trial. Secondly, since the p-values for the effect of the intervention was similar in the group comparisons and the prediction models, it implies that the unexpected negative effect of the intervention is not bound to individuals of a specific gender, smoking habit, socialising habit, exercise level or high/low BMI. It does nor rule out the possibility of individuals with other features, not measured in this study, being prone to experience negative effects of the studied intervention.

**Supplemental table 1. Internal validation of prediction models based on binary logistic regression.**

| Outcome (dependent variable)^1^ | Area Under Curve ROC | Nagelkerke R Square |
| --- | --- | --- |
| Change in Physical Functioning^2^ | 0.60 (p=0.17, 95% CI 0.46-0.74) | 0.04 |
| **Change in Role-Physical^2^** | **0.71 (p=0.012, 95% CI 0.55-0.87)** | **0.14** |
| Change in Bodily Pain^2^ | 0.57 (p=0.32, 95% CI 0.43-0.70) | 0.018 |
| **Change in General Health^2^** | **0.63 (p=0,036, 95% CI 0.51-0.76)** | **0.084** |
| Change in Vitality^2^ | 0.60 (p=0.12, 95% CI 0.48-0.71) | 0.045 |
| **Change in Social Functioning^2^** | **0.71 (p=0.010, 95% CI 0.56-0.87)** | **0.13** |
| **Change in Role-Emotional^2^** | **0.73 (p=0.0014, 95% CI 0.62-0.85)** | **0.18** |
| **Change in Mental Health ^2^** | **0.71 (p=0.0010, 95% CI 0.60-0.82)** | **0.17** |
| **Change in Physical Component Summary^3^** | **0.66 (p=0.011, 95% CI 0.55-0.77)** | **0.13** |
| **Change in Mental Component Summary^4^** | **0.74 (p=0.00011, 95%CI 0.63-0.85)** | **0.21** |
| **Change in Exercise level^5^** | **0.83 (p=1.8x10^-5^, 95% CI 0.72-0.94)** | **0.37** |
| Change in Socialising Habits^6^ | 0.61 (p=0.21, 95% CI 0.46-0.75) | 0.035 |

^1^ Models with statistically significant Area Under Curve values > 0.5 are bolded. Significance was set at p>0.05. The Outcome (dependent variable) was coded as follows: worsening and no change is coded as 0. Improvement is coded as 1.

^2^ Change in the sub scale/dimension of SF-36 Health Survey.

^3^ Change in the general dimension based on sum scores of physical functioning, role-physical, bodily pain and general health.

^4^ Change in general dimension based on sum scores of vitality, social functioning, role-emotional and mental health.

^5^ Change in Exercise level.

^6^ Change in socialising habits in leisure time.

**Supplemental table 2. Prediction models for outcome at 12 months where internal validation supports the model.1, 2**

|  | RP^3^ | | GH^4^ | | SF^5^ | | RE^6^ | | MH^7^ | | PCS^8^ | | MCS^9^ | | EL^10^ | |
| --- | --- | --- | --- | --- | --- | --- | --- | --- | --- | --- | --- | --- | --- | --- | --- | --- |
|  | P^11^ | aOR^12^ | P^11^ | aOR^12^ | P^11^ | aOR^12^ | P^11^ | aOR^12^ | P^11^ | aOR^12^ | P^11^ | aOR^12^ | P^11^ | aOR^12^ | P^11^ | aOR^12^ |
| Female gender | 0.37 | 0.54  (0.14-2.1) | 0.88 | 0.92  (0.33-2.6) | 0.092 | 3.5 (0.81-15) | 0.14 | 2.6  (0.72-9.1) | 0.40 | 0.64  (0.22-1.8) | 0.87 | 1.1  (0.38-3.1) | 0.48 | 1.5  (0.50-4.4) | **0.031** | **7.2  (1.2-43)** |
| Increasing smoking^13^ | 0.98 | 0.99  (0.57-1.7) | 0.48 | 1.2  (0.77-1.8) | 0.16 | 1.4  (0.86-2.4) | **0.035** | **1.7  (1.0-2.7)** | 0.10 | 1.4  (0.94-2.2) | 0.11 | 0.70  (0.45-1.1) | 0.071 | 1.5  (0.97-2.4) | 0.93 | 0.98  (0.55-1.7) |
| Socialising Habits^14^ | 0.24 | 1.7  (0.70-4.2) | 0.55 | 0.83  (0.44-1.5) | 0.16 | 0.54  (0.23-1.3) | 0.23 | 0.63  (0.29-1.3) | 0.14 | 0.62  (0.32-1.2) | 0.18 | 1.6 (0.81-3.0) | **0.049** | **0.50  (0.25-1.0)** | 0.99 | 1.0  (0.38-2.6) |
| Exercise Level^15^ | 0.23 | 1.7  (0.71-4.3) | 0.93 | 0.97  (0.46-2.0) | 0.24 | 1.8  (0.68-4.5) | 0.38 | 1.5  (0.62-3.5) | 0.15 | 1.8  (0.82-3.8) | 0.14 | 0.55  (0.25-1.2) | 0.11 | 1.9  (0.86-4.2) | **0.0024** | **0.10  (0.021-0.43)** |
| BMI | 0.20 | 1.1 (0.94-1.3) | 0.75 | 0.98  (0.85-1.1) | 0.61 | 1.0  (0.88-1.2) | 0.31 | 1.1  (0.93-1.3) | 0.52 | 0.95  (0.82-1.1) | 0.47 | 1.1  (0.92-1.2) | 0.74 | 0.98  (0.85-1.1) | 0.47 | 0.93  (0.78-1.1) |
| Group^16^ | 0.38 | 0.56  (0.15-2.0) | **0.047** | **0.38  (0.15-1.0)** | 0.21 | 0.46  (0.14-1.6) | ***0.052*** | ***0.32  (0.10-1.0)*** | 0.19 | 0.53  (0.20-1.4) | 0.29 | 0.60  (0.24-1.5) | **0.033** | **0.35  (0.13-0.92)** | 0.22 | 2.5  (0.59-10) |

^1^ This table only shows prediction models where the ROC-analysis shows they were better than pure chance.

^2^ Statistically significant p-values and effect sizes are bolded in this table. The level of significance was set to 0.05.

^3^ Role-physical sub scale/dimension from the SF-36 Health Survey. OR>1 is a desired change

^4^ General health sub scale/dimension from the SF-36 Health Survey. OR>1 is a desired change

^5^ Social functioning sub scale/dimension from the SF-36 Health Survey. OR>1 is a desired change

^6^ Role-emotional sub scale/dimension from the SF-36 Health Survey. OR>1 is a desired change

^7^ Mental health sub scale/dimension from the SF-36 Health Survey. OR>1 is a desired change

^8^ Physical component summary general dimension from the SF-36 Health Survey. OR>1 is a desired change

^9^ Mental component summary general dimension from the SF-36 Health Survey. OR>1 is a desired change

^10^ Exercise level at 12 months (worsening or no change coded as 0 and improvement coded as 1)

^11^ p-value.

^12^ Adjusted odds ratio (95% confidence interval for adjusted odds ratio).

^13^ Odds ratio for an increase of one step in smoking at baseline: 1=Never smoked, 2=Ex smoker, 3= Occasional smoker, 4=Smoking daily.

^14^ Odds ratio for an increase of one step in socialising habits at baseline: 1=Never socialise, 2=Rarely socialise, 3= Socialise several times per months, 4=Socialise several times per week

^15^ Odds ratio for an increase of one step in exercise level at baseline: 1=Sedentary, 2=Light exercise, 3=Regular exercise, 4=Tough exercise or competitive sports

^16^ Odds ratio for belonging to the health coach group (active intervention coded as 1) as compared to the control group (coded as 0)

**Supplemental table 3. Comparison between p-values obtained in the group comparisons
with p-values found in the prediction models**^1^

|  | =============== Group comparison =============== | | | Prediction model |
| --- | --- | --- | --- | --- |
| Change in | Per Protocol analysis | Complete Case analysis | Intention to treat analysis |  |
| Role physical (RP) | 0.29-0.46 | 0.040-0.091 | 0.036-0.086 | 0.38 |
| General Health (GH) | 0.034-0.083 | 0.0050-0.029 | 0.0055-0.025 | 0.047 |
| Role emotional (RE) | 0.038-0.047 | 0.027-0.032 | 0.034-0.040 | 0.052 |
| Mental component Summary (MCS) | 0.042-0.090 | 0.034-0.088 | 0.033-0.073 | 0.033 |

^1^ This table shows p-values for the effect of the intervention. It compares p-values obtained in the group comparison (table 4) to the corresponding p-values obtained in the prediction model (supplemental table 2). Comparison is only included if there are significant, or near significant, p-values in the group comparison and the corresponding prediction model is better than pure chance.
